# Nasopharyngeal Testing among Healthcare Workers (HCWs) of a Large University Hospital in Milan, Italy during Two Epidemic Waves of COVID-19

**DOI:** 10.3390/ijerph18168748

**Published:** 2021-08-19

**Authors:** Agnese Comelli, Dario Consonni, Andrea Lombardi, Giulia Viero, Massimo Oggioni, Patrizia Bono, Sara Colonia Uceda Renteria, Ferruccio Ceriotti, Davide Mangioni, Antonio Muscatello, Alessandra Piatti, Angela Cecilia Pesatori, Silvana Castaldi, Luciano Riboldi, Alessandra Bandera, Andrea Gori

**Affiliations:** 1 Infectious Diseases Unit, Foundation IRCCS Ca’ Granda Ospedale Maggiore Policlinico, 20122 Milan, Italy; andrea.lombardi@policlinico.mi.it (A.L.); giulia.viero@unimi.it (G.V.); davide.mangioni@unimi.it (D.M.); antonio.muscatello@policlinico.mi.it (A.M.); alessandra.bandera@policlinico.mi.it (A.B.); andrea.gori@policlinico.mi.it (A.G.); 2 Epidemiology Unit, Foundation IRCCS Ca’ Granda Ospedale Maggiore Policlinico, 20122 Milan, Italy; dario.consonni@policlinico.mi.it (D.C.); angela.pesatori@policlinico.mi.it (A.C.P.); 3 Department of Pathophysiology and Transplantation, University of Milan, 20122 Milan, Italy; 4 Clinical Laboratory, Foundation IRCCS Ca’ Granda Ospedale Maggiore Policlinico, 20122 Milan, Italy; massimo.oggioni@policlinico.mi.it (M.O.); patrizia.bono@policlinico.mi.it (P.B.); sara.ucedarenteria@policlinico.mi.it (S.C.U.R.); ferruccio.ceriotti@policlinico.mi.it (F.C.); 5 Medical Direction, Foundation IRCCS Ca’ Granda Ospedale Maggiore Policlinico, 20122 Milan, Italy; alessandra.piatti@policlinico.mi.it; 6 Department of Clinical Sciences and Community Health, University of Milan, 20122 Milan, Italy; 7 Department of Biomedical Sciences for Health, University of Milan, 20122 Milan, Italy; silvana.castaldi@policlinico.mi.it; 8 Quality Unit, Foundation IRCCS Ca’ Granda Ospedale Maggiore Policlinico, 20122 Milan, Italy; 9 Occupational Health Unit, Foundation IRCCS Ca’ Granda Ospedale Maggiore Policlinico, 20122 Milan, Italy; luciano.riboldi@policlinico.mi.it; 10 Centre for Multidisciplinary Research in Health Science (MACH), University of Milan, 20122 Milan, Italy

**Keywords:** COVID-19, SARS-CoV-2, healthcare operators, reinfection, nasopharyngeal swab

## Abstract

Background: since October 2020, a second SARS-CoV-2 epidemic wave has hit Italy. We investigate the frequency of positive nasopharyngeal swabs among HCWs during the two waves and the association with occupation and demographic characteristics. Methods: this is a retrospective, observational study conducted in a large university hospital in Milan, Northern Italy. We defined two epidemic waves: 1st (February 2020–July 2020) and 2nd (August 2020–January 2021). Occupational and demographic characteristics of HCWs who underwent nasopharyngeal swabs for SARS-CoV-2 were collected. Results: in the 1st wave, 242 positive subjects (7.2%) were found among 3378 HCWs, whereas in the 2nd wave, the positive subjects were 545 out of 4465 (12.2%). In both epidemic waves positive NPSs were more frequent among HCWs with health-related tasks and lower among students (*p* < 0.001). However, in the 2nd wave, workers engaged in non-health-related tasks had a peak of 20.7% positivity. Among 160 positive HCWs in the 1st wave who were tested again in the 2nd wave, the rate of reinfection based on SARS-CoV2 RNA cycle quantification value was 0.6%. Conclusions: during the 2nd epidemic wave, we confirmed a significant impact of COVID-19 among HCWs. The rise of infection rate among HCWs seems to reflect the increasing spread of SARS-CoV-2 among the overall population.

## 1. Introduction

The novel severe acute respiratory syndrome coronavirus 2 (SARS-CoV-2) was first detected in Wuhan (China) at the end of 2019 following the appearance of a cluster of severe pneumonia cases [1]. The virus rapidly spread worldwide and the World Health Organization (WHO) declared the new coronavirus disease 2019 (COVID-19) as a pandemic in early March 2020.

**Figure 1:**
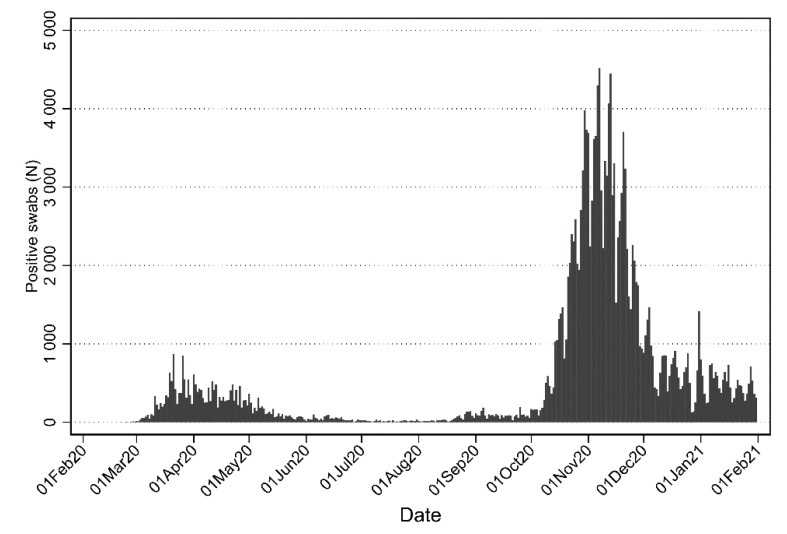
Number of positive nasopharyngeal tests per day in the Metropolitan City of Milan, Italy from 24 February 2020 to 31 January 2021. Data source: https://github.com/pcm-dpc/ (accessed on 10 August 2021).

Since late February 2020, Italy was one of the most affected country in Europe. The Metropolitan City of Milan (Lombardy region) was heavily affected by COVID-19 epidemic, with 205,939 positive cases from 24 February 2020 to 28 February 2021 among a population of 3.2 million people [2]. As of 31 July 2020 the cumulative number of positive subjects was 24,926, with a peak in April 2020 (10,426 cases) [3]. The epidemic wave got worse in the following months. Even considering that a larger number of swabs was performed daily, there was a more than six-fold increase in the positivity rates, with a total of 164,197 positive cases from August 2020 to January 2021, with a peak in November 2020 (82,565 cases) (Figure 1) [3]. 

The SARS-CoV-2 pandemic deeply affected health care workers (HCWs), requesting their unprecedented efforts in all countries around the world. In Italy, more than 125,000 HCWs were infected in the first year of pandemic [2]. Health care personnel was employed on the frontline to guarantee patients care and consequently exposed to a higher risk in comparison to the general population [4]. Several studies conducted in hospitals located in the Milan area, reported a range of SARS-CoV-2 seroprevalence of 5.4–7.6% among HCWs [5,6].

We previously evaluated the prevalence of SARS-CoV-2 infection among HCWs employed in a university and research hospital in the center of Milan, Italy, from late February 2020 to 31 March 2020: we found that 8.8% of HCWs tested because of compatible symptoms or because they were a contact of a confirmed case of COVID-19, resulted positive to nasopharyngeal swab (NPS) [7]. Some months later, we observed in the same hospital a similar rate of infection from February to July 2020: 8.1% [8].

In the present study we extended data collection and analyzed NPS results during the second epidemic wave of COVID-19 which occurred in the fall 2020–winter 2021. As in the previous report, we assessed the frequency of positive tests among HCW and we evaluated the association with occupational and demographic characteristics. We concluded the analysis at the end of January 2021, because in that month a COVID-19 vaccination campaign was started.

## 2. Materials and Methods

### 2.1. Study Design, Population, and Design

This is a retrospective, observational study conducted at the Foundation IRCCS Ca’ Granda Ospedale Maggiore Policlinico, in Milan, Northern Italy during the SARS-CoV-2 pandemic. We collected occupational and clinical characteristics of all consecutive HCWs who underwent nasopharyngeal swabbing for the detection of SARS-CoV-2 infection.

Since no worker tested positive for SARS-CoV-2 in July 2020, we arbitrarily defined two epidemic waves: 1st wave (24 February 2020 to 1 July 2020) and 2nd wave (from 1 August 2020 to 31 January 2021).

Both in the 1st and 2nd waves, symptomatic HCWs or those at risk for infection, defined as contact with a patient or another HCW with documented SARS-CoV-2 infection, were tested according to local clinical practice (only nasopharyngeal swab). In the 2nd wave, in addition, HCWs employed in clinical wards underwent systematic testing in the context of mandatory surveillance screening every 2–4 weeks (according to internal surveillance guidelines).

A specific code to identify mandatory surveillance swabs was established only from 1 December 2020, while the reason of swabbing was not available in October and November.

HCWs were classified into physicians (including residents), nurses and midwives, healthcare assistants, health technicians (including biologists, radiology and laboratory technicians, psychologists and other health technicians), clerical workers and technicians, and students.

For those HCWs with a suspected reinfection, defined as a positive NPS occurring more than 90 days since the first positive NPS, a phone interview was conducted to collect missing information [9].

Standard PPE (Personal Protective Equipment) used by healthcare professionals when caring for COVID-19 patients or during activities of daily work living have complied with WHO rules [10]. Internal guidelines and practical demonstrations via video recordings were regularly distributed to HCWs in accordance with updated international recommendations.

The study was conducted in accordance with the Helsinki Declaration.

### 2.2. Laboratory Procedures

For viral detection two different methods were used. The first one employed Seegene Inc reagents (Seoul, Korea). RNA extraction and PCR plate preparation were performed with STARMag Universal Cartridge kit on Nimbus/starlet instrument (Hamilton, Agrate Brianza, Italy) and amplification with Allplex^®^ 2019-nCoV assay. The second one employed a GeneFinder^®^ COVID-19 Plus RealAmp Kit (OSANG Healthcare, Anyangcheondong-ro, Dongan-gu, Anyang-si, Gyeonggi-do, Korea) on ELITech InGenius^®^ instrument (Torino, Italy). Both assays identify the virus by multiplex rRT-PCR targeting three viral genes (E, RdRP and N). SARS-CoV-2 serology was performed with LIAISON SARS-CoV-2 S1/S2 IgG test on LIAISON XL (DiaSorin, Saluggia, Italy).

Cycle quantification value (Cq) was calculated for every single rRT-PCR and, accordingly with the available literature [11,12], a cut off of 35 Cq was used.

### 2.3. Statistical Analysis

We merged demographic files with laboratory files containing NPS results. Then we divided the dataset in the two waves. Within each period we graphed the number of NPSs over time. Then for each worker we determined the date of the first positive test (if any) and described the trend of positive subjects over time. Exact (Clopper-Pearson) 95% confidence intervals (CI) of proportions were calculated. We compared frequency of workers with at least one positive NPS according to selected variables using the chi-squared test. Finally, we calculated the proportion of re-infections in the second wave among subjects who tested positive in the first wave and were re-tested in the second one. Statistical analysis was performed with Stata 16 (StataCorp. 2019).

## 3. Results

**Figure 2:**
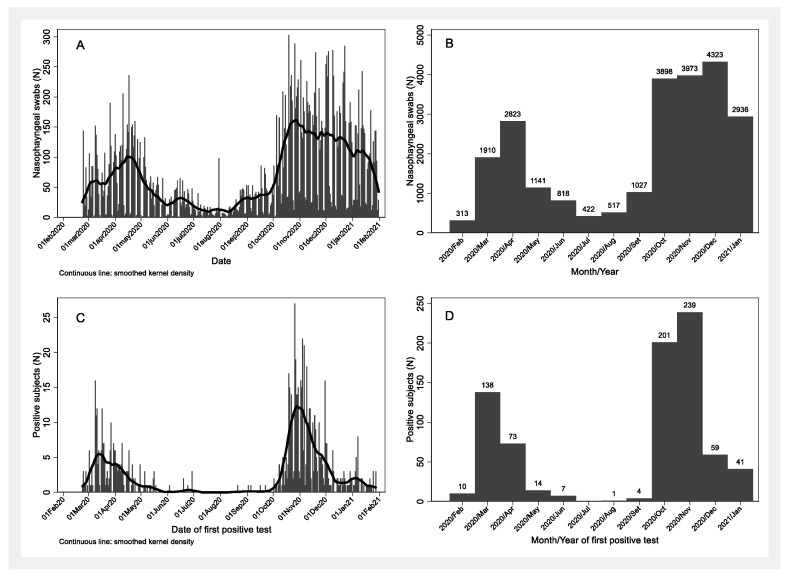
Number of nasopharyngeal tests per day (**A**), per month (**B**) and number of positive subjects per day (**C**) and per month (**D**) among healthcare workers during the first (February to July 2020) and the second epidemic wave (August 2020 to January 2021) in a research and teaching hospital in Milan, Italy.

Overall, from 24 February 2020 to 31 January 2021, 24,101 NPSs were performed: with a peak in April 2020 and in December 2020 (Figure 2A,B). Overall, in the 1st wave there were 242 positive subjects out of 3378 tested HCWs (7.2%, 95% CI: 6.3–8.1%), while in the 2nd wave the positive workers were 545 out of 4465 (12.2%, 95% CI: 11.3–13.2%). The number of subjects with a positive rRT-PCR for SARS-CoV-2 rose rapidly and then declined slowly in both waves, but in the 2nd wave the decrease was followed by a slight rise in January 2021 (Figure 2C). The number of positive subjects peaked in March 2020 (*n* = 138) and in November 2020 (*n* = 239) (Figure 2D).

Surveillance program on HCWs started on October 2020 and at least 2604 out of 4465 (58.3%) HCWs were tested because of routine surveillance in the 2nd wave (those identified by a specific code during December 2020–January 2021). Consequently, less than 1861 individuals were tested because of symptoms/close contact in October 2020–January 2021. At least 3.3% (87/2604) infected HCWs were detected thanks to the mandatory surveillance campaign.

In the 1st wave the positivity rate was higher in men (*p* = 0.001), whether in the 2nd wave identical proportions were found. No major variations of positivity according to age were found in the 1st wave, while in the 2nd wave there was an increasing trend with increasing age. In both epidemic waves positive NPSs were more frequent among HCW with health-related tasks and lower among students (*p* < 0.001). However, in the 2nd wave, workers engaged in non-health-related tasks had a peak of 20.7% positivity (Table 1).

Among the 160 HCWs who tested positive in the 1st wave and who repeated NPS during 2nd wave, nine (5.6%, 95% CI: 2.6–10.4%), three men and six women, were found to be newly positive in the 2nd wave (Table 2). All were first found positive in March-April 2020, all of them had a negative NPS after a median of 17 days (inter-quartile range 15–31) and they were again found positive in the period from November 2020 to January 2021. The median time elapsed between the first positive test in the 1st wave and the first positive test in the 2nd wave was 235 days (inter-quartile range 220–253).

During 1st wave, all but one HCWs with suspected reinfection underwent NPS because of COVID-19 related symptoms. By contrast, in the 2nd wave seven out of nine of them tested positive during routine surveillance.

Among the nine HCWs with suspected reinfection, eight underwent serologic test (with a median interval from the 1st positive NPS of 55 days). Five out of eight (62.5%) showed a positive titer of anti-SARS-CoV-2 spike antibodies before the supposed second infection, including the two workers symptomatic during the supposed re-infection on 2nd wave.

We reviewed microbiologic data: patient 1 was the only subject whose NPS resulted positive for SARS-CoV-2 gene E, N and RdRP and with a low cycle quantification (Cq) value (20.8). He was seronegative after the first infection. All the other HCWs’ NPS resulted positive only for gene E with high Cq value (Table 2). Based on these data, the reinfection rate drops to 1/160 (0.6%, 95%CI 0.2–3.4%).

## 4. Discussion

In a large university research and teaching hospital in the center of Milan, in the most affected region in Italy, we confirmed a significant impact of the COVID-19 epidemic among HCWs. The impact was larger in the 2nd wave, where the proportion of positive workers was 12.2–70% higher than in the 1st wave (7.2%).

A reasonable explanation to the rise of infection rate among HCWs is that they were involved, like the surrounding population, to an increasing spread of SARS-CoV-2 over the months. During the 2nd wave the lockdown rules were laxer and international and national travel were possible allowing easier circulation of the virus than before when the rules in force were extremely harsh.

Conversely, thanks to the growing knowledge about SARS-CoV-2 transmission gained during the pandemic progression, the recommendations about PPE applied during the 2nd wave were certainly more evidence-based and a lot of work was completed to update the HCWs of our hospital through practical demonstrations and publication of updated procedures.

All things considered, although healthcare workers have a high risk of exposure, this was offset by the extensive use of PPE which ultimately resulted in a similar, or at least not increased, risk of infection among healthcare workers compared to the general population.

Furthermore, it should be noted that in Lombardy region, while the sadly famous Province of Bergamo, was heavily hit during in the 1st wave [13], the Milan area faced a much higher spread in the 2nd wave (Figure 1).

Lastly, the appearance of virus variants in late 2020, mainly the lineage B.1.1.7, characterized by higher transmission rate, could have contributed to a new pandemic surge [14].

Other groups in Europe reported a broad range of infection rate among HCWs during the 1st wave: from 2.6% to 24.9% [15,16,17,18,19,20,21], conversely, few studies have been published on the evolution of SARS-CoV-2 infection among European HCWs between the 1st and 2nd wave [22,23].

Looking at previous studies carried out at the Foundation IRCCS Ca’ Granda Ospedale Maggiore Policlinico, the proportion of subjects positive at NPS during the 1st wave (7.2%) matches the seroprevalence calculated among the total population of HCWs employed during the pandemic (7.6%) [6]. Consequently, we can assume that the targeted swabbing during the 1st wave was successful in detecting the HCWs infected by SARS-CoV-2. Moreover, we previously showed (Lombardi et al.) that the exposure within the community probably represented an important mode of HCW infection during 1st wave [6]. The same conclusion is offered by other groups [24,25,26].

During the 2nd wave, the virus circulation has risen in the community thanks to a less tight lockdown and to a wider possibility of regional/national mobility [27]. To reinforce the hypothesis that the spread of SARS-CoV-2 in hospitals is likely to reflect what is happening in the community, we observed that HCW engaged in non-health-related tasks had a peak of 20.7% positivity in the 2nd wave whereas they accounted for 4% during 1st wave. Furthermore, if we look at the number of SARS-CoV-2 cases in the Metropolitan City of Milan in relation to its inhabitants, the positive rate was 0.7% at the end of June 2020 (24,379/3,265,327) and it rose to 6.0% at the end of January 2021 (189,123/3,156,694) [3,28].

The two-waves analysis allowed us to identify potential reinfection among HCWs, an issue of particular interest because of their high risk of exposure and frequent testing regardless of clinical manifestations. In our cohort, the rate of suspected re-infections was 5.6% (9/160) with 62.5% of them with a positive serology tested before the second NPS. The majority of HCWs with suspected reinfection was tested in the context of surveillance screening (during 2nd wave).

Using the Cq value as a tool to distinguish suspected and proved reinfection, our rate drops to 0.6%. Virologists largely agree that samples of SARS-CoV-2 RNA with a quantification cycle value of 35 or greater did not produce virus isolation in cell cultures suggesting that those positive results represent shedding of nonviable virus [11].

Our adjusted reinfection rate is in agreement with studies from Denmark, UK, US and Qatar that reported rare reinfection occurring in fewer than 1% of all COVID-19 cases [22,23,29,30].

Lumley et al. reported 2 reinfections out of 1177 seropositive HCWs (0.2%) versus 2% among seronegative subjects. In contrast to our study, they do not refer to a previous NPS but to a prior positive serology, thus not excluding the possibility of false positive serologies explaining the following suspected reinfections [23].

In Denmark, Hansen et al. followed up 658 positive HCWs and only 8 subjects (1.2%) were reinfected. Unfortunately, they did not perform further analysis to confirm reinfection against prolonged shedding or false results [22].

Similar to our observations, Abu-Raddad et al. initially registered a 0.7% of reinfection rate but this rate dropped to 0.1% when verified by a second method (viral genome sequencing) [30].

Even if the persistence of SARS-CoV-2 spike protein-specific antibodies after 3–8 months post infection was described by several reports [31,32], data on their ability to predict a robust and persistent T cell memory are conflicting [32,33], leaving open the possibility of re-infection also among seropositive subjects. Anyway, the majority of these studies, included ours, cannot exclude that those interpreted as reinfections may, on the contrary, be infections with variants of SARS-CoV-2 unrecognized at that time.

This study was based on routinely collected data. Thus, we could provide a description of SARS-CoV-2 spread in the hospital in a rapid and efficient way. On the other hand, use of routine data has some drawbacks. The hospital demographic file for students, residents, and temporary workers was incomplete with a certain number of workers with missing or ill-defined jobs. Moreover, in the 2nd wave a high proportion of NPS were performed as HCWs surveillance whether in the 1st wave we recorded only NPS done in the context of contact tracing/symptoms. For this reason, we did not formally compare the positive rate of the two waves. In addition, because of late introduction of a specific code for identify NPS done as screening, we cannot be sure about the proportion of positive subject tested because of contact tracing/symptoms.

Despite these limitations the present study is one of the few works that provide information on SARS-CoV-2 (re-)infection among HCWs comparing the two epidemic waves. Moreover, even if not studied in depth, it suggests the possibility of reinfection, even if rare. Of course, to better understand the real impact of reinfection, immunological and genotypical studies are needed, in particular at the present moment with the spread of SARS-CoV-2 variants.

Unfortunately, no structured testing strategies was implemented: e.g., not all the 242 positive HCWs in the 1st wave were systematically retested in the 2nd wave.

## 5. Conclusions

We documented a significant impact of the SARS-CoV-2 epidemic in a large research and teaching hospital in Milan, that probably reflects the spread of infection in the surrounding population. We expect a substantial improvement in the following months thanks to the start of large vaccination campaigns among both HCWs and the overall population.

## Figures and Tables

**Table 1:** Association between selected variables and frequency of subjects with positive SARS-CoV-2 nasopharyngeal tests among healthcare workers during the first (February to July 2020) and the second epidemic wave (August 2020 to January 2021) in a research and teaching hospital in Milan, Italy.

	1st Wave			2nd Wave		
Variable	HCWs	Positives		HCWs	Positives	
	N	N	%	N	N	%
All	3378	242	7.2	4465	545	12.2
Gender						
Women	2357	147	6.2	3140	383	12.2
Men	1021	95	9.3	1325	162	12.2
*p*-value			0.001			0.98
Age (years)						
<30	796	49	6.2	1242	109	8.8
30–39	810	62	7.6	1136	143	12.6
40–49	617	48	7.8	795	109	13.7
50–59	842	62	7.4	939	128	13.6
60+	313	21	6.7	353	56	15.9
*p*-value			0.73			<0.001
Occupation						
Physicians, including residents	959	83	8.6	1221	134	11.0
Nurses, midwives	1092	81	7.4	1285	192	14.9
Healthcare assistants	274	29	10.6	335	50	14.9
Health technicians ^a^	359	30	8.4	516	69	13.4
Clerical workers, technicians	252	10	4.0	376	78	20.7
Students	326	5	1.5	576	12	2.1
Missing/ill-defined	116	4	3.4	156	10	6.4
*p*-value			<0.001			<0.001

^a^ Including biologists, radiology and laboratory technicians, psychologists, other health technicians.

**Table 2:** Description of the nine cases suspected of reinfection.

Patient n.	Age	Sex	Occupation	1st Positive NPS (Date)	Symptoms at 1st Positive NPS	2nd Positive NPS (Date)	Symptoms at 2nd Positive NPS	Cq of SARS-CoV2 Gene E ^a^	Time Elapsed between the Two Positive NPS (Days)	Serologic Test ^b^ (Date)	Days from Positive NPS to Serology Test	Serologic Test Result (AU/mL) ^c^
1	48	M	Healthcare assistant	20/03/20	Asymptomatic	19/11/20	Asymptomatic ^d^	20.8 ^e^	244	14/05/20	55	Negative
2	54	M	Nurse, midwife	20/03/20	Cough, fatigue	12/01/21	Asymptomatic ^d^	>35	298	19/05/20	60	Positive (85.2)
3	31	M	Physician, including resident	03/04/20	Fatigue	10/11/20	Asymptomatic ^d^	>35	221	05/06/20	63	Negative
4	31	F	Nurse, midwife	20/03/20	Cough	01/12/20	Asymptomatic ^d^	>35	256	19/05/20	60	Positive (21.6)
5	60	F	Nurse, midwife	15/04/20	Fatigue, dyspnoea, ageusia/anosmia, fever, headache	26/11/20	Diarrhoea	>35	225	06/05/20	21	Positive (42.2)
6	61	F	Nurse, midwife	03/04/20	Cough, fatigue, ageusia/anosmia, fever, headache	09/12/20	Asymptomatic ^d^	>35	250	18/05/20	45	Positive (85.2)
7	34	F	Clerical worker, technician	14/04/20	Cough, ageusia/anosmia, rhinorrhoea,	18/11/20	Asymptomatic ^d^	>35	218	18/05/20	34	Negative
8	29	F	Student	14/04/20	ageusia/anosmia GI discomfort, headache	19/11/20	Asymptomatic ^d^	>35	219	NA		
9	57	F	Nurse, midwife	03/04/20	Cough, fatigue, GI discomfort, fever	24/11/20	Fatigue, headache, myalgia	>35	235	27/05/20	54	Positive (73.5)

^a^ Cq: cycle quantification value. It refers to the number of cycles needed to amplify viral RNA to reach a detectable level. ^b^ performed with LIAISON SARS-CoV-2 S1/S2 IgG test on LIAISON XL (DiaSorin, Saluggia, Italy). ^c^ A test was considered positive when the value observed was equal to or above 15 AU/mL. ^d^ performed as HCW surveillance. ^e^ patient 1 was the only subject whose NPS resulted positive for SARS-CoV-2 gene E, N and RdRP. All the other HCWs’ NPS were positive only for gene E. NA: not available.

## Data Availability

Not applicable.
